# An Empirically Derived Definition of Metabolically Healthy Obesity Based on Risk of Cardiovascular and Total Mortality

**DOI:** 10.1001/jamanetworkopen.2021.8505

**Published:** 2021-05-07

**Authors:** Anika Zembic, Nathalie Eckel, Norbert Stefan, Julia Baudry, Matthias B. Schulze

**Affiliations:** 1 Department of Molecular Epidemiology, German Institute of Human Nutrition Potsdam-Rehbruecke, Nuthetal, Germany; 2 German Center for Diabetes Research, Neuherberg, Germany; 3 Institute of Nutritional Science, University of Potsdam, Potsdam, Germany; 4 Institute of Diabetes Research and Metabolic Diseases of the Helmholtz Center München, University of Tübingen, Tübingen, Germany; 5 Department of Internal Medicine IV, University Hospital of Tübingen, Tübingen, Germany; 6 Sorbonne Paris North University, the National Institute for Health and Medical Research, the National Research Institute for Agriculture, Food and Environment, the National Conservatory of Arts and Crafts and the Nutritional Epidemiology Research Team, Epidemiology and Statistics Research Centre, University of Paris, Bobigny, France

## Abstract

**Importance:**

People classified by a priori definitions as having metabolically healthy obesity have frequently been found to be at increased risk of mortality, compared with individuals with metabolically healthy normal weight, suggesting these definitions may be insufficient.

**Objectives:**

To systematically derive a new definition of metabolic health (MH) and investigate its association with cardiovascular disease (CVD) mortality and total mortality.

**Design, Setting, and Participants:**

In a cohort study using data from the third National Health and Nutrition Examination Survey (NHANES-III), a representative survey using complex multistage probability sampling, anthropometric factors, biomarkers, and blood pressure (BP) associated with total and CVD mortality among participants with obesity were identified with Cox proportional hazards regression. Area under the receiver operating characteristic was calculated to identify predictive factors for mortality to be used to define MH, cutoff levels were determined by the Youden index, and the findings were validated through comparison with the independent UK Biobank cohort, a population-based prospective study. All nonpregnant participants in the databases aged 18 to 75 years with no history of CVD, body mass index greater than or equal to 18.5, and who fasted 6 or more hours before examination in NHANES-III were included; participants in the UK Biobank cohort who did not have blood measurements were excluded. The study was conducted from 2015 to 2020.

**Exposures:**

Body mass index and MH were defined by the new definition and compared with 3 a priori definitions.

**Main Outcomes and Measures:**

Cardiovascular disease mortality and total mortality.

**Results:**

Within the NHANES-III (n = 12 341) cohort, mean (SD) age was 41.6 (29.2) years, 50.7% were women, and mean follow-up was 14.5 (2.7) years. Within the UK Biobank (n = 374 079) cohort, mean (SD) age was 56.2 (8.1) years, 55.1% were women, and mean follow-up was 7.8 (1.0) years. Use of blood pressure (BP)-lowering medication (hazard ratio [HR] for CVD mortality, 2.41; 95% CI, 1.50-3.87 and total mortality, 2.05; 95% CI, 1.47-2.84), diabetes, and several continuous factors were associated with mortality. Of all significant continuous factors, the combination of systolic BP and waist-to-hip ratio showed the highest area under the receiver operating characteristic (CVD mortality: 0.775; 95% CI, 0.770-0.781; total mortality: 0.696; 95% CI, 0.694-0.699). Thus, MH was defined as systolic BP less than 130 mm Hg, no BP-lowering medication, waist-to-hip ratio less than 0.95 for women and less than 1.03 for men, and no self-reported (ie, prevalent) diabetes. In both cohorts, metabolically healthy obesity was not associated with CVD and total mortality compared with metabolically healthy normal weight. For NHANES-III, the hazard ratio was 0.68 (95% CI, 0.30-1.54) for CVD mortality and 1.03 (95% CI, 0.70-1.51) for total mortality. For UK Biobank, the hazard ratio was 1.17 (95% CI, 0.81-1.69) for CVD mortality and 0.98 (95% CI, 0.87-1.10) for total mortality. Regardless of body mass index, all metabolically unhealthy groups displayed increased risks.

**Conclusions and Relevance:**

This newly proposed definition of MH may identify a subgroup of people with obesity without increased risk of mortality and stratify risks in people who are overweight or normal weight.

## Key Points

**Question:**

Can a definition of metabolic health be empirically derived that allows to stratify risk of mortality in people with obesity?

**Findings:**

In this study including data from 386 420 individuals across 2 cohorts, a new definition of metabolically healthy obesity was established. People with a phenotype defined as metabolically unhealthy using this definition had significantly higher hazard ratios for cardiovascular disease mortality and total mortality irrespective of body mass index category, and people with phenotypes defined as having metabolically healthy obesity displayed no increased risk.

**Meaning:**

A new definition of metabolic health identified in this study may allow to stratify risk of mortality not only in people with obesity but also in people with overweight and normal weight.

## Teaser

This cohort study formulates a new definition of metabolically healthy obesity and examines its use in estimation of cardiovascular disease mortality and total mortality.

## Introduction

A 2018 study has shown that obesity, defined as body mass index (BMI) of 30 or more (calculated as weight in kilograms divided by height in meters squared), is associated with cardiometabolic diseases and increased mortality.^1^ However, not all people with obesity have an increased risk of cardiometabolic diseases. This subgroup is considered as having metabolically healthy obesity (MHO) and their counterpart, individuals with metabolically unhealthy obesity (MUHO), might have an excess risk for adverse outcomes.^2^ Debate on this phenotype relates to the lack of a uniform definition to identify metabolic health (MH).^3^ Previously used definitions related to MH are frequently based on the absence of metabolic syndrome or absence of insulin resistance (eg, assessed by homeostatic model assessment of insulin resistance [HOMA-IR]).^4^ In theory, cardiovascular disease (CVD) and mortality risk of individuals with MHO should be similar compared with individuals who have metabolically healthy normal weight (MHNW). However, meta-analyses^5,6^ and studies in large-scale cohorts^7,8^ observed increased risks for CVD mortality and total mortality for individuals with MHO despite the absence of metabolic syndrome. Although some studies indicate that stricter definitions of MH based on metabolic syndrome (absence of all components excluding waist circumference), may allow identification of a low-risk obesity group,^6^ a recent study does not support this notion.^8^ Using previous definitions and criteria may not be sufficient to identify an obese subgroup not at increased risk.

An alternative approach to using a priori definitions of MH is to explore risk factors for mortality among people with obesity and empirically derive a new definition. To our knowledge, this has not been applied systematically before; therefore, we aimed to establish a new definition for MH based on the mortality follow-up of the third National Health and Nutrition Examination Survey (NHANES-III). We evaluated the association of this new definition with CVD mortality and total mortality, validated it in an independent cohort (UK Biobank), and compared it with commonly used a priori definitions in both cohorts.

## Methods

The data analyses of the 2 cohort studies were conducted from 2015 to 2020. The NHANES-III study was approved by the National Center for Health Statistics and the UK Biobank study was approved by the North West Multicenter Research Ethics Committee. This study followed the Strengthening the Reporting of Observational Studies in Epidemiology (STROBE) reporting guideline for cohort studies.

### NHANES-III

NHANES-III, a representative survey with complex multistage probability sampling design, was conducted between 1988 and 1994 in the US. People aged 2 to 5 years, 60 years and older, and those who were Mexican Americans or African American were oversampled.^9^ Baseline data assessments included a personal interview and physical examinations in 33 199 participants. The NHANES-III protocol was approved by the National Center for Health Statistics. The participants gave written informed consent before participation.^9^ Our analyses included all nonpregnant participants aged 18 to 75 years with no history of CVD, BMI greater than or equal to 18.5, and fasted for 6 or more hours before the examination (n = 12 341).

Mortality data, including the underlying causes of death until 2006, was used and classified by *International Statistical Classification of Disease, 10th Revision* (*ICD-10*) codes.^10^ Both total and CVD mortality (*ICD-10* codes I00-I78) were considered as outcomes.

### UK Biobank

The UK Biobank is a large, population-based prospective study with 502 506 participants aged 40 to 69 years. Baseline assessment (22 assessment centers, 2006-2010) included electronic signed informed consent, a self-completed questionnaire, personal interview, physical measurements, and blood collection.^11^ The North West Multicenter Research Ethics Committee approved the study.^12^

In contrast to NHANES-III, UK Biobank participants were not instructed to fast before assessment.^12^ Because no fasting blood levels were necessary for the main analysis (n = 374 079), we made exclusions identical to those with NHANES-III plus exclusion of missing covariates and conducted a separate analysis (n = 41 431) in which we additionally excluded participants who fasted for less than 6 hours. This research was conducted using the UK Biobank resource under application number 50426. Mortality follow-up data were available via linkage with national death registries until 2016 for UK Biobank, and we used identical *ICD-10* codes to define the outcomes as in NHANES-III.^12^

### Measurements

Participants were categorized into BMI groups: normal weight (18.5-24.9), overweight (25.0-29.9), and obesity (≥30.0). Anthropometric and blood pressure (BP) measurements were conducted comparably in both cohorts (eTable 1 in the Supplement). Blood samples were analyzed for triglyceride, total cholesterol, high-density lipoprotein cholesterol, glucose, and hemoglobin A_1c_ levels.^12,13^ In addition, we used measurements of C-reactive protein, insulin, γ-glutamyltransferase, and alanine aminotransferase levels from NHANES-III.^13^ Insulin sensitivity was estimated with quantitative insulin sensitivity check index, because it exhibited stronger correlations with the hyperinsulinemic-euglycemic clamp than HOMA-IR in a meta-analysis from 2014.^14^ Race/ethnicity and prevalent diabetes were self-reported in both cohorts; current medication was self-reported in the UK Biobank and containers were presented at the interview in NHANES-III.^12,15^

### Statistical Analysis

We applied multiple imputation to missing data on outcome, exposure, and confounder variables for the NHANES-III sample (eTable 2 in the Supplement).^16^ We used the Markov Chain Monte Carlo method to create 10 data sets with monotone missing patterns and monotone methods to impute the remaining missing values. Rubin’s rules were used to combine the results.^17^

To identify anthropometric and metabolic parameters associated with CVD or total mortality, we calculated Cox proportional hazard regression models in a subsample of NHANES-III participants with obesity (using proc surveyphreg to account for the survey design). We used age as the underlying time scale and adjusted for the following known confounders (self-reported with predefined categories to choose from): sex (male, female), race/ethnicity (non-Hispanic white, non-Hispanic black, Mexican-American, other), educational level (≤8, 9-12, ≥13 years of school completed), income (<$20 000 or≥$20 000 per year), marital status (married, previously, never), smoking status (never, former, current), alcohol consumption (0, 1-10, >10 drinks per month), and physical activity (never, 1-8, >8 times per month). Similar to definitions of metabolic syndrome,^18^ we aimed to identify elevated risk factor levels as components of an MH definition either when participants were receiving drug treatment or, among nontreated individuals, on measured risk factor levels. Thus, for continuous factors that showed significant associations with CVD mortality or total mortality, we calculated areas under the receiver operating characteristic (AUROC) curve in a subsample of participants with obesity without BP-lowering, glucose-lowering, and lipid-lowering medication (n = 2511) to evaluate which factors discriminate best between at risk and not at risk. We analyzed each factor separately and then in combined models, starting with the metabolic factor with the highest AUROC and extending by those with the next highest AUROC. Factors remained in the model when their inclusion led to a significant improvement in AUROC. We used Youden index to determine statistically optimal cutoff levels.

We then compared relative mortality risks in subgroups stratified by BMI and MH using aforementioned adjustments. We defined MH using our new definition, as well as 3 a priori definitions:

1. Absence of metabolic syndrome according to National Cholesterol Education Adult Treatment Panel-III criteria. Individuals are considered healthy if 2 or fewer criteria are present: waist circumference, 102 cm (men) or greater than 88 cm (women); BP greater than or equal to 130/85 mm Hg or using BP-lowering medication; triglyceride level greater than or equal to 150 mg/dL or using lipid-lowering medication; high-density lipoprotein cholesterol level less than 40 mg/dL (men) or less than 50 mg/dL (women); and fasting glucose level greater than or equal to 110 mg/dL or prevalent diabetes.^18^
2. HOMA-IR (healthy if <2.5 [arbitrary unit of measure]).
3. Strict definition. Individuals are considered healthy if all of the following criteria are absent: BP greater than or equal to 130/85 mm Hg or using BP-lowering medication; fasting glucose level greater than or equal to 100 mg/dL and hemoglobin A_1c_ level greater than or equal to 5.7% (39 mmol/mol) or using glucose-lowering medication; triglyceride level greater than or equal to 150 mg/dL, total cholesterol level greater than or equal to 240 mg/dL, or high-density lipoprotein cholesterol level less than 40 mg/dL (men) or less than 50 mg/dL (women) or using lipid-lowering medication.

To compare our new definition with these definitions, we used models with mutual adjustments for our and the other definitions, assuming that a definition that is superior in distinguishing between at-risk and not-at-risk individuals would identify other population subgroups and its associations with mortality would remain unaffected when adjusted for other definitions. To address typical sources of bias in obesity/mortality analyses^19^ we performed 4 sensitivity analyses: exclusion of ever smokers, deaths within the first 2 years of follow-up, participants older than 60 years, and changing the reference BMI category to 20.0 to 22.49. In addition, we repeated the analysis stratified by sex and CVD mortality subtype (coronary heart disease and stroke mortality).

We applied our new definition in the UK Biobank, using identical Cox proportional hazards regression models of associations between subgroups of BMI and MH status and CVD and total mortality and adjustments as used in NHANES-III, with additional adjustment for assessment center. We attempted to group covariates into categories as similar as possible to the NHANES-III analysis: sex (male, female), race/ethnicity (White, mixed, Asian, Black, Chinese, other), educational level (university/college [UK degree, similar to US graduate school], A levels [UK degree, similar to US high school diploma], O levels/GCSE [UK degree, similar to US grade 10], CSE [UK degree awarded until 1987 equivalent to GSCE], NVQ [technical training]), income (<£18 000 or≥£18 000 per year [approximately $25 000]), marital status (married and living together, living with another family member, living alone), smoking status (never, former, current), alcohol consumption (never, special occasions, 1-3 times per month, 1-2 times per week, 3-4 times per week, daily or almost daily), and physical activity (never, ≤20 minutes, and >20 minutes of vigorous activity per week). We performed the following analyses with identical models as conducted in NHANES-III: comparison of MH defined by our new definition with MH defined by National Cholesterol Education Adult Treatment Panel-III criteria and the strict definition, sensitivity analyses, and stratified analyses as described above. Because insulin levels were not measured in the UK Biobank it was not possible to compare MH defined by HOMA-IR with the other definitions.

All analyses were performed in SAS, version 9.4, Enterprise Guide 7.1 (SAS Institute Inc). Significance tests were 2-tailed and *P* values <.05 were considered statistically significant. Analysis within NHANES-III was conducted in 2015 and the UK Biobank was conducted in 2020.

## Results

The NHANES-III participants (n = 12 341) had a mean (SD) age of 41.6 (29.2) years and mean BMI of 27.2; 50.7% of the participants were women and 49.3% were men. The UK Biobank participants (n = 374 079) had a mean age of 56.2 (8.1) years and a mean BMI of 27.4; 55.1% were women and 44.9% men. During a mean (SD) follow-up of 14.5 (2.7) years in the NHANES-III cohort, 1758 deaths from all causes and 594 deaths associated with CVD occurred; during the mean (SD) 7.8 (1.0)-year follow-up in the UK Biobank cohort, 12 950 deaths from all causes and 2162 deaths associated with CVD occurred. In both cohorts, participants with obesity were less likely to have higher educational levels, more likely to be categorized in the lowest income group, and more likely to be physically inactive, compared with normal-weight and overweight participants. The UK Biobank cohort consisted of older, less ethnically diverse participants, and the proportion of never drinkers and current smokers was considerably smaller compared with the NHANES-III cohort. Generally, the confounder structure was similar in both cohorts (Table 1).

**Table 1.** Baseline Characteristics by BMI Category

Characteristic	Normal weight (BMI, 18.5-24.9)	Overweight (BMI, 25.0-29.9)	Obesity (BMI, ≥30)
**NHANES-III (n = 12 341)**^a^			
No. (%)	4888 (39.6)	4217 (34.2)	3236 (26.2)
BMI, mean (SD)	22.2 (1.78)	27.1 (3.31)	34.7 (10.2)
Age, mean (SD), y	38.0 (32.1)	43.8 (28.2)	44.3 (26.1)
Sex, %			
Women	55.4	40.2	57.3
Men	44.6	59.8	42.7
Ethnicity, %			
Non-Hispanic White	77.6	76.4	72.9
Non-Hispanic Black	9.07	10.5	14.0
Mexican American	4.31	6.32	6.19
Other	9.00	6.75	6.92
Years of school completed, %			
≥13 y	47.3	41.9	34.7
9-12 y	45.9	47.8	54.1
≤8 y	6.80	10.3	11.2
Income, %			
<$20 000	29.5	28.8	34.0
Marital status, %			
Married or living as married	61.0	72.0	68.8
Previously married	14.4	12.6	18.2
Never married	24.7	15.3	13.0
Smoking status, %			
Never	47.5	45.5	46.2
Former	20.4	27.5	29.7
Current	32.2	27.0	24.1
Alcohol consumption, %			
0 drinks/mo	37.0	40.2	51.5
1-10 drinks/mo	32.3	30.9	31.4
>10 drinks/mo	30.7	28.8	17.2
Physically active, %			
Never	28.8	36.5	44.2
1-8 times/mo	22.7	23.1	23.9
>8 times/mo	48.5	40.4	31.8
**UK Biobank (n = 374 079)**			
No. (%)	125 598 (33.6)	160 124 (42.8)	88 357 (23.6)
BMI, mean (SD)	22.9 (1.5)	27.3 (1.4)	33.9 (3.9)
Age, mean (SD), y	55.5 (8.2)	56.7 (8.1)	56.4 (7.9)
Sex, %			
Female	65.6	47.6	53.7
Male	34.4	52.4	46.3
Race/ethnicity, %			
White	95.5	95.2	94.6
Mixed	0.62	0.52	0.59
Asian	1.77	1.92	1.56
Black	0.76	1.27	2.20
Chinese	0.60	0.23	0.07
Other	0.77	0.83	0.93
Educational level, % ^b^			
University/college	39.9	32.0	25.3
A levels	12.2	11.1	10.7
O levels/GCSE	20.6	21.6	22.5
CSE	4.80	5.46	6.61
NVQ	4.84	7.01	7.79
Other professional	4.72	5.24	5.59
Income, %			
<18 000 GBP	16.3	17.9	22.4
Marital status/living arrangements, %			
Married and living together	72.7	75.6	70.4
Living with other family member	8.81	7.53	9.68
Living alone	18.5	16.9	19.9
Smoking status, %			
Never	59.5	54.4	52.4
Former	29.4	35.6	38.0
Current	11.1	10.1	9.59
Alcohol consumption, %			
Never	22.7	21.6	15.6
Special occasions only	24.9	24.8	19.4
1-3 Times per month	25.4	26.4	26.5
1-2 Times per week	10.3	10.5	13.6
3-4 Times per week	9.74	9.95	15.4
Daily or almost daily	6.95	6.85	9.49
Physical activity, vigorous, %			
Never	34.9	38.8	49.0
≤20 min/wk	12.5	12.2	12.3
>20 min/wk	52.5	49.0	41.7

Abbreviations: BMI, body mass index (calculated as weight in kilograms divided by height in meters squared); NHANES-III, National Health and Nutrition Examination survey.

^a^ NHANES-III data weighted to the US population.

^b^ University/college (UK degree, similar to US graduate school), A levels (UK degree, similar to US high school diploma), O levels/GCSE (UK degree, similar to US grade 10), CSE (UK degree awarded until 1987 equivalent to GSCE), NVQ (technical training).

Anthropometric and metabolic factors significantly associated with both CVD and total mortality in NHANES-III were waist circumference (hazard ratio [HR] per SD, CVD mortality: 1.40; 95% CI, 1.18-1.66; total mortality: 1.35; 95% CI, 1.19-1.52), waist-to-hip ratio (WHR) (HR per SD, CVD mortality: 1.37; 95% CI, 1.08-1.75; total mortality: 1.29; 95% CI, 1.10-1.51), quantitative insulin sensitivity check index (HR per SD, CVD mortality: 0.74; 95% CI, 0.58-0.94; total mortality: 0.74; 95% CI, 0.64-0.86), use of BP-lowering medication (HR per SD, CVD mortality: 2.41; 95% CI, 1.50-3.87; total mortality: 2.05; 95% CI, 1.47-2.84), and prevalent diabetes (HR per SD, CVD mortality: 1.80; 95% CI, 1.02-3.18; total mortality: 1.88; 95% CI, 1.27-2.78). In addition, systolic BP was significantly associated with CVD mortality (HR per SD, 1.32; 95% CI, 1.07-1.62), and BMI (HR per SD, 1.20; 95% CI, 1.06-1.36), fasting glucose (HR per SD, 1.15; 95% CI, 1.02-1.30), hemoglobin A_1c_, (HR per SD, 1.13; 95% CI, 1.01-1.27), C-reactive protein (HR per SD, 1.18; 95% CI, 1.04-1.34), and γ-glutamyltransferase (HR per SD, 1.14; 95% CI, 1.06-1.23) levels were significantly associated with total mortality (Table 2). We retained the categorical factors prevalent diabetes and use of BP-lowering medication and subsequently calculated AUROCs for all significant continuous factors (Table 3) within NHANES-III. Systolic BP showed the highest predictive ability for both outcomes (CVD mortality: AUROC, 0.775; 95% CI, 0.770-0.781; total mortality: AUROC, 0.696; 95% CI, 0.694-0.699). Of the remaining variables, WHR improved the AUROC significantly, but only for total mortality (0.640; 95% CI, 0.639-0.642). Statistically significant optimal cutoffs for systolic BP were 125 (Youden index, 0.460) and 124 mm Hg (Youden index, 0.320) for CVD and total mortality (eTable 3 in the Supplement). However, owing to the proximity of these levels to the well-established cutoff of 130 mm Hg for pre-hypertension, we decided to adhere to that established cutoff. When evaluating WHR, AUROCs were higher for CVD mortality than for total mortality; hence, we used the WHR cutoff for CVD mortality. Accordingly, we constructed the following criteria for MH:

**Table 2.** Metabolic Markers for CVD and Total Mortality Among Participants With Obesity (NHANES-III, n = 3236)

Variable	HR (95% CI)^a^	
	CVD mortality	Total mortality
Body mass index, per SD	1.20 (0.99-1.44)	1.20 (1.06-1.36)^b^
Waist circumference, per SD	1.40 (1.18-1.66)^b^	1.35 (1.19-1.52)^b^
Waist-to-hip ratio, per SD	1.37 (1.08-1.75)^b^	1.29 (1.10-1.51)^b^
Systolic blood pressure, per SD^c^	1.32 (1.07-1.62)^b^	1.16 (0.98-1.38)
Diastolic blood pressure, per SD^c^	1.17 (0.83-1.65)	0.94 (0.75-1.18)
Triglycerides, per SD^d^	1.07 (0.85-1.35)	1.14 (0.99-1.30)
Total cholesterol, per SD^d^	1.14 (0.88-1.48)	1.00 (0.86-1.16)
HDL cholesterol, per SD^d^	0.98 (0.74-1.31)	0.84 (0.70-1.01)
Fasting glucose, per SD^e^	1.02 (0.72-1.44)	1.15 (1.02-1.30)^b^
Hemoglobin A_1c_, per SD^e^	1.24 (0.97-1.57)	1.13 (1.01-1.27)^b^
QUICKI, per SD^e^	0.74 (0.58-0.94)^b^	0.74 (0.64-0.86)^b^
C-reactive protein, per SD	1.20 (0.99-1.45)	1.18 (1.04-1.34)^b^
Alanine aminotransferase, per SD	1.06 (0.77-1.45)	1.16 (0.97-1.39)
γ-Glutamyltransferase, per SD	1.08 (0.96-1.23)	1.14 (1.06-1.23)^b^
Lipid-lowering medication (yes vs no)	1.35 (0.53-3.42)	1.26 (0.70-2.25)
BP-lowering medication (yes vs no)	2.41 (1.50-3.87)^b^	2.05 (1.47-2.84)^b^
Diabetes (yes vs no)	1.80 (1.02-3.18)^b^	1.88 (1.27-2.78)^b^

Abbreviations: BMI, body mass index; BP, blood pressure; CVD, cardiovascular disease; HDL, high density lipoprotein; HR, hazard ratio; NHANES-III, National Health and Nutrition Examination Survey-III; QUICKI, quantitative insulin sensitivity check index.

^a^ HRs and 95% CIs were weighted to the US population and adjusted for age, sex, race/ethnicity, educational level, income, marital status, smoking status, alcohol consumption, and physical activity.

^b^ Significant association at *P* < .05.

^c^ Exclusion of participants with BP-lowering medication (n = 2628).

^d^ Exclusion of participants with lipid-lowering medication (n = 3178).

^e^ Exclusion of participants with glucose-lowering medication (n = 3066).

**Table 3.** Factors Associated With CVD and Total Mortality for Combinations of Factors Among Participants With Obesity (NHANES-III, n = 2511)^a^

Variable	CVD mortality		Total mortality	
	AUROC (95% CI)	Change in AUROC	AUROC (95% CI)	Change in AUROC
Waist circumference	0.684 (0.681 to 0.688)	NA	0.633 (0.630 to 0.635)	NA
Waist-to-hip ratio	0.702 (0.697 to 0.706)	NA	0.640 (0.639 to 0.642)	NA
BMI	0.516 (0.513 to 0.520)	NA	0.518 (0.516 to 0.519)	NA
Systolic BP	0.775 (0.770 to 0.781)	NA	0.696 (0.694 to 0.699)	NA
QUICKI	0.553 (0.545 to 0.561)	NA	0.551 (0.546 to 0.556)	NA
Fasting glucose	0.574 (0.562 to 0.586)	NA	0.605 (0.601 to 0.610)	NA
Hemoglobin A_1c_	0.644 (0.638 to 0.651)	NA	0.608 (0.603 to 0.612)	NA
C-reactive protein	0.553 (0.547 to 0.560)	NA	0.530 (0.526 to 0.534)	NA
γ-Glutamyltransferase	0.618 (0.603 to 0.632)	NA	0.565 (0.557 to 0.574)	NA
SBP	0.775 (0.770 to 0.781)	[Reference]	0.696 (0.694 to 0.699)	[Reference]
SBP + WHR	0.802 (0.797 to 0.806)	0.026 (−0.009 to 0.062)	0.720 (0.718 to 0.722)	0.024 (0.001 to 0.047)
SBP + WHR	NA	[Reference]	NA	[Reference]
SBP + WHR + WC	0.804 (0.799 to 0.808)	0.002 (−0.013 to 0.017)	0.724 (0.722 to 0.726)	0.004 (−0.005 to 0.013)
SBP + WHR + HbA1c	0.805 (0.801 to 0.809)	0.004 (−0.007 to 0.014)	0.722 (0.720 to 0.724)	0.002 (−0.003 to 0.007)
SBP + WHR + GGT	0.805 (0.800 to 0.810)	0.003 (−0.003 to 0.010)	0.720 (0.719 to 0.722)	0.000 (−0.001 to 0.002)
SBP + WHR + Glucose	0.802 (0.798 to 0.807)	0.001 (−0.002 to 0.003)	0.721 (0.719 to 0.723)	0.001 (−0.002 to 0.004)
SBP + WHR + QUICKI	0.801 (0.796 to 0.805)	−0.001 (−0.007 to 0.005)	0.721 (0.719 to 0.723)	0.001 (−0.003 to 0.004)
SBP + WHR + CRP	0.806 (0.801 to 0.810)	0.004 (−0.008 to 0.017)	0.725 (0.723 to 0.726)	0.004 (−0.005 to 0.013)
SBP + WHR + BMI	0.802 (0.798 to 0.807)	0.001 (−0.010 to 0.011)	0.722 (0.720 to 0.724)	0.002 (−0.005 to 0.008)

Abbreviations: AUROC, area under the receiver operating characteristic; BMI, body mass index; CRP, C-reactive protein; CVD, cardiovascular disease; GGT, γ-glutamyltransferase; NA, not applicable; NHANES-III, National Health and Nutrition Examination Survey-III; QUICKI, quantitative insulin sensitivity check index; SBP, systolic blood pressure; WC, waist circumference; WHR, waist-to-hip ratio.

^a^ Exclusion of participants using blood pressure–lowering, glucose-lowering, or lipid-lowering medication.

1. systolic BP less than 130 mm Hg and no use of BP-lowering medication,
2. WHR less than 0.95 (women) and less than 1.03 (men), and
3. no prevalent diabetes.

**Figure 1.**
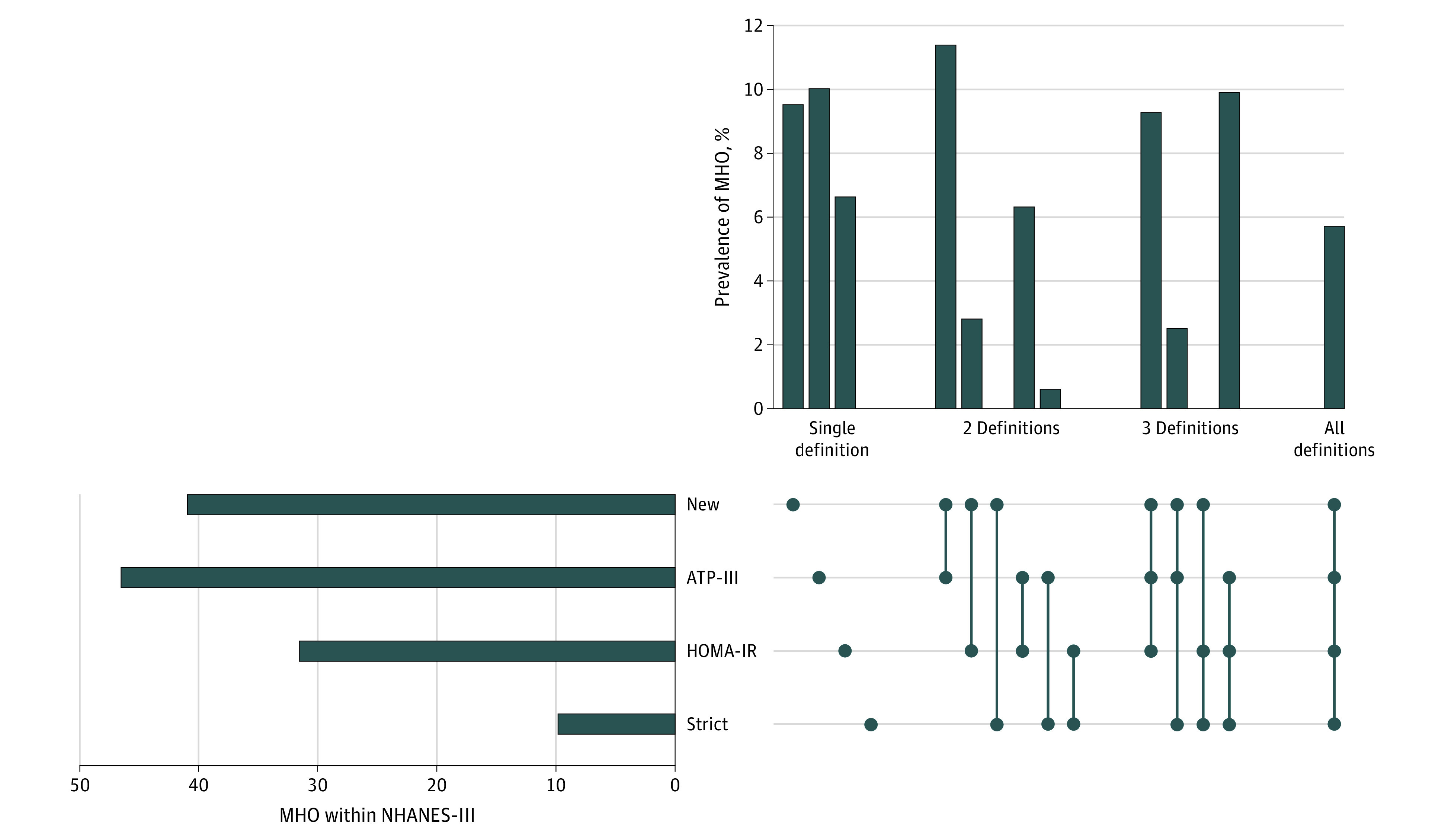
Weighted Prevalence and Overlap of Metabolically Healthy Obesity (MHO) Identified by Different Definitions Within the National Health and Nutrition Examination Survey-III (NHANES-III) (n = 12 341) ATP-III indicates Adult Treatment Panel-III; HOMA-IR, homeostatic model assessment of insulin resistance.

The proportion of participants with obesity who did not fulfill criteria was 42.2% for criterion 1, 33.9% for criterion 2, and 6.58% for criterion 3 in NHANES-III and, in the UK Biobank, was 77.7% for criterion 1, 14.1% for criterion 2, and 9.9% for criterion 3. According to the new definition, 41.2% of participants with obesity were metabolically healthy within NHANES-III and 19.3% of participants with obesity were metabolically healthy within the UK Biobank. The proportion of MHO by the other 3 definitions in NHANES-III was 9.9% (strict definition), 31.7% (HOMA-IR), and 46.7% (National Cholesterol Education Adult Treatment Panel-III criteria). Only 5.7% of NHANES-III participants had MHO according to all 4 definitions (Figure 1). More details on the frequency of covariates in each group of MH and BMI can be found in eTable 4 in the Supplement.

**Figure 2.**
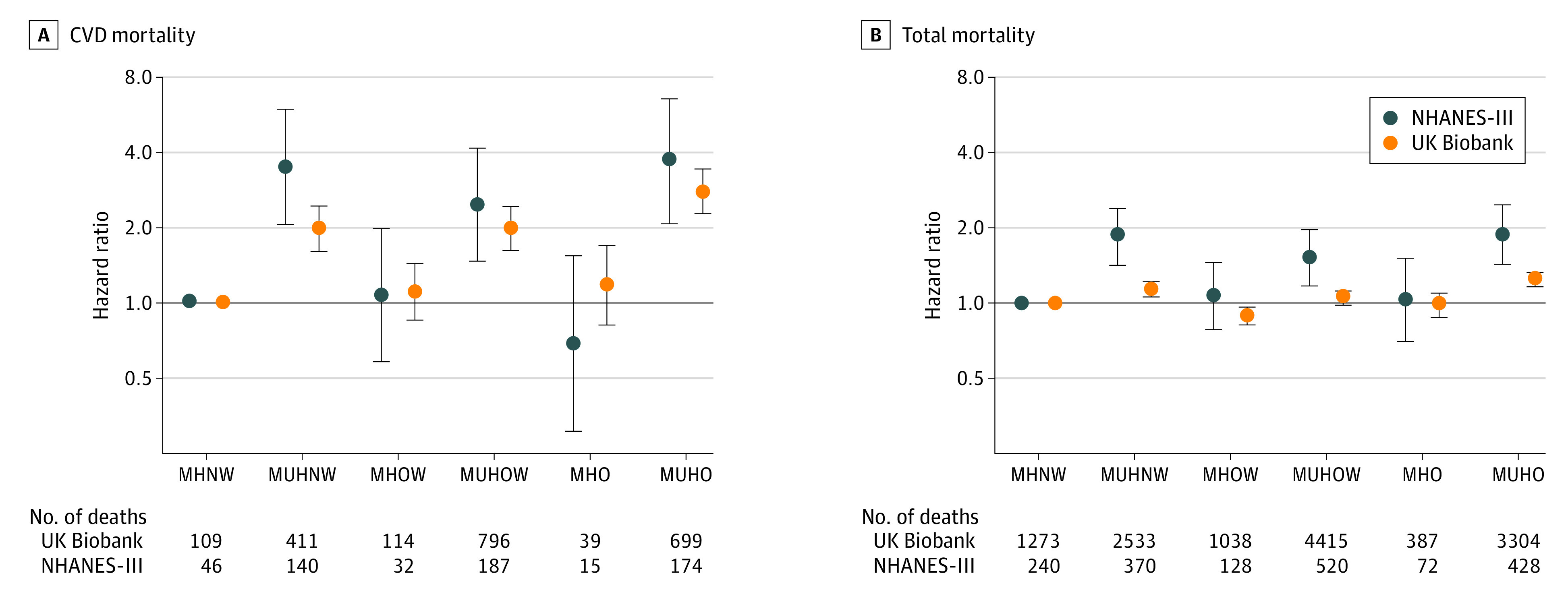
Risk of Mortality in Subgroups of Body Mass Index and Metabolic Health Using a New Definition Hazard ratios (95% CIs) adjusted for age, sex, race/ethnicity, educational level, income, marital status, smoking status, alcohol consumption, physical activity, and UK Biobank assessment center. National Health and Nutrition Examination Survey-III (NHANES-III) cohort, n = 12 341; UK Biobank cohort, n = 374 079. CVD indicates cardiovascular disease; MHNW, metabolically healthy normal weight; MHO metabolically healthy obese; MHOW, metabolically healthy overweight; MUHNW, metabolically unhealthy normal weight; MUHO, metabolically unhealthy obese; and MUHOW, metabolically unhealthy overweight.

Cross-classifying participants by BMI and MH defined by our new definition yielded increased adjusted risks for CVD mortality for all individuals classified as unhealthy, compared with individuals with MHNW independently of BMI categories in both cohorts. Conversely, CVD mortality of individuals with MHO was not significantly increased compared with MHNW participants (NHANES-III: hazard ratio, 0.68; 95% CI, 0.30-1.54; UKB: hazard ratio, 1.17; 95% CI, 0.81-1.69) (Figure 2). These associations remained stable when we adjusted for the other 3 definitions (eFigure 1 in the Supplement).

When MH was defined by the other definitions, similar associations could be observed only in some instances (eFigure 2 in the Supplement). Metabolic health defined by National Cholesterol Education Adult Treatment Panel-III criteria revealed differing risk groups within the UK Biobank; however, almost none of the groups displayed increased risks in NHANES-III, especially when adjusted for the new definition. When MH was defined by the strict definition, most participants were classified as unhealthy within both cohorts, which led to the healthy groups being small and, in the MHO group in the UKB, even too small for analyses (no cases observed). Defining MH according to HOMA-IR was only possible within NHANES-III. Increased risks could be observed in the MUHNW and the MUHO groups for both total and CVD mortality, but not in the MUHOW group. For all 3 definitions, the risk estimates were lower when adjusted for our new definition.

When total mortality was investigated, similar risk patterns were observed in both cohorts: individuals classified as MHO by the new definition were not at increased risk compared with MHNW participants (NHANES-III: HR, 1.03; 95% CI, 0.70-1.51; UK Biobank: HR, 0.98; 95% CI, 0.87-1.10), but individuals classified as metabolically unhealthy had an increased risk, irrespective of BMI category (Figure 2). However, the risk estimates were considerably smaller in the UK Biobank. These associations remained when adjusted for the other definitions (eFigure 3 in the Supplement). When using the other 3 methods to define MH, results similar to those for CVD mortality were observed (eFigure 4 in the Supplement).

All sensitivity analyses yielded qualitatively similar results in both cohorts (eTable 5 and eTable 6 in the Supplement). We observed similar risks for women and men with MHO (eTable 7 in the Supplement). Individuals with MHO were not at increased risk for both coronary heart disease and stroke mortality compared with MHNW participants (eTable 8 in the Supplement). Furthermore, we subdivided participants with obesity into obesity class 1 (BMI, 30.0-34.9), 2 (35.0-39.9), and 3 (≥40) and repeated the main analysis within the UK Biobank (eTable 9 in the Supplement). The analysis confirmed higher risks for both outcomes in unhealthy groups. Metabolically healthy obesity was not associated with increased risks compared with MHNW for obesity classes 1 and 2 (CVD mortality, obesity class 1: HR, 1.00; 95% CI, 0.65-1.52; obesity class 2: HR, 0.83; 95% CI, 0.31-2.26). However, for obesity class 3, MHO was associated with higher risks for CVD mortality compared with MHNW (HR, 6.34; 95% CI, 3.43-6.16). When defining MH with only 2 of the 3 criteria of our definition, omitting 1 criterion at a time, we observed relatively similar increases in HRs for CVD mortality for MHO (without diabetes criterion: HR, 1.31; 95% CI, 0.92-1.84; without WHR criterion: HR, 1.28; 95% CI 0.92-1.80; without BP criterion: HR, 1.32; 95% CI, 1.15-1.51), supporting the importance of all 3 criteria (eTable 10 in the Supplement).

## Discussion

This systematic assessment of mortality data and various anthropometric and metabolic factors yielded a simple definition to categorize participants with obesity as metabolically healthy or unhealthy. This definition classified more than 40% of individuals with obesity as MHO within the NHANES-III population, a representative sample of the US adult population, and 20% within the UK Biobank; this group was not at increased risk for CVD and total mortality compared with MHNW individuals. Conversely, individuals classified as metabolically unhealthy were at increased risk compared with MHNW participants, independent of BMI category in both cohorts. The results were robust after accounting for typical sources of bias for the associations between obesity and mortality. The risks of both outcomes were almost equally increased in participants with MUHNW as in MUHO in both cohorts, indicating that our new definition may also be helpful to detect an increased risk in lean people.

Prospective studies on CVD and mortality risks of MHO provided inconsistent results.^3,4^ The use of varying definitions for MH might be responsible for the observed heterogeneity.^20^ Often, the absence of metabolic syndrome or insulin resistance has been used to identify MHO, however, with differing criteria and cutoffs. When the results from individual studies using a variety of definitions based on metabolic syndrome or HOMA-IR and risk for CVD events and total mortality were summarized in meta-analyses, individuals with MHO were still at increased risk compared with individuals with MHNW, especially in the long term.^5,6^ In the present study, MHO defined by either of these 2 definitions showed inconsistent results among both cohorts, and the associations were not independent of our new definition; however, our definition was associated with the risk of mortality independently of other definitions. This finding suggests that our definition better distinguishes between at-risk and not-at-risk individuals. Our results, however, also indicate that our definition may only be able to identify a low-risk MHO phenotype among individuals with a BMI less than 40. Higher mortality was observed for people with severe obesity (BMI ≥40), irrespective of MH.

Our definition has substantial differences from common versions of metabolic syndrome definitions. Waist circumference as a marker for abdominal obesity was replaced by WHR, which has the advantage of less-pronounced correlations with BMI^21,22^ and the additional consideration of hip circumference. Although waist circumference estimates visceral fat, which is a risk factor for metabolic disorders and CVD, waist circumference also estimates subcutaneous abdominal fat, which is considered to have less-detrimental effects on metabolism.^23^ In contrast, hip circumference is a proxy of lower body fat, which might have protective effects on metabolism.^23,24,25^ In support of stronger predictive power of WHR compared with waist circumference, WHR was more associated with mortality than waist circumference in individuals with high BMI.^21^

Because measures of dyslipidemia were not significantly associated with mortality in our study, our new definition does not include criteria for dyslipidemia. Although this lack of inclusion seems surprising considering the well-known association between dyslipidemia and increased risk of death and CVD,^26,27^ associations between total cholesterol level and vascular mortality are weaker among participants with obesity compared with other BMI categories.^28^

Furthermore, our definition only classifies individuals as MH who fulfill all 3 criteria, whereas using the absence of metabolic syndrome to define MH allows up to 2 risk factors to be present. Our study supports findings from 2 studies that found that the absence of all metabolic syndrome criteria (without the waist circumference criterion) identifies people with obesity who are not at increased risk for total and CVD mortality.^29,30^ Nevertheless, in our analysis the association of this strict definition with total mortality was attenuated when adjusted for our new definition. The simultaneous absence of all metabolic syndrome criteria classifies a small fraction of individuals with obesity as MH, whereas our exploratory definition identified a considerably larger proportion of people with obesity not at increased risk for total and CVD mortality.

Our results for MH and total mortality, using metabolic syndrome criteria and HOMA-IR to define MH, are in agreement in direction of effect with a previous analysis in the NHANES-III population, using the same duration of follow-up; however, effect estimates differ quantitatively.^31^

### Strengths and Limitations

To our knowledge, this is the first study that systematically assessed multiple cardiometabolic risk factors for a possible definition for the MHO phenotype. Strengths of this study are the prospective design, long follow-up, large sample sizes, and validation in an independent cohort. Limitations are lack of data on changes in body weight and metabolic factors owing to the original design of NHANES-III. Thus, we were unable to investigate the outcomes associated with changes in MH status, which have been reported to substantially alter CVD risk.^3,8^ Furthermore, body fat distribution might differ according to race/ethnicity and our proposed WHR cutoff might not be suitable for all populations, especially Asian populations, which were underrepresented in both cohorts.^32,33^ In addition, although NHANES-III is a representative survey, the UK Biobank consists of volunteers not representative of the UK population^34^ and has a higher mean age, which might explain the difference in proportions of MHO participants between the 2 cohorts. Another limitation is that, although the C index might be better suited for survival data than area under the receiver operating characteristic, the C index was not available for the surveyphreg procedure. Also, we cannot rule out that alternative variable selection methods may have resulted in different criteria. Still, we validated the utility of our definition by evaluating associations with mortality, also in comparison with other definitions and by repeating the analyses in an independent cohort.

## Conclusions

In this study, we developed a new definition to identify individuals with MHO, based on self-reported diabetes, use of BP medication, systolic BP, and WHR, which can be used in both clinical and research settings. Our results suggest that people with MHO classified by this definition are not at increased risk for CVD or total mortality. Metabolically unhealthy individuals have a substantially higher risk, which is not explained by conventional definitions of MH. Thus, our new definition may be important not only to stratify risk of mortality in people with obesity, but also in people with overweight and normal weight.

## Notes

Supplement. **eTable 1.** Baseline Assessment Within the Third National Health and Nutrition Examination Survey (NHANES-III) and the UK Biobank **eTable 2.** Summary of Missing Data for NHANES-III (n = 17 742) **eTable 3.** Cutoffs at Maximal Youden Index Among Obese Participants (NHANES-III n = 2511)* **eTable 4.** Baseline Characteristics of UK Biobank Participants in Groups of Metabolic Health and BMI (n = 374 079) **eTable 5.** Sensitivity Analysis of CVD Mortality **eTable 6.** Sensitivity Analysis of Total Mortality **eTable 7.** Stratified Analysis by Sex **eTable 8.** Stratified Analysis by CVD Type **eTable 9.** Adjusted HRs and 95% CIs of CVD and Total Mortality in Subgroups Classified by BMI Categories Extended by Obesity Class 1 to 3 and MH Defined by Our New Definition (UK Biobank n = 374 079) **eTable 10.** Adjusted HRs and 95% CIs of CVD and Total Mortality in Subgroups Classified by BMI Categories and MH Defined by a) Our New Definition, b) New Definition Without the Diabetes Criterion, c) New Definition Without the WHR Criterion, d) New Definition Without the Blood Pressure Criterion (UK Biobank n = 374 079) **eFigure 1.** Adjusted HRs and 95% CIs for Risk of CVD Mortality in Subgroups Classified by BMI Categories and MH Defined by a) Our New Exploratory Definition b) New Definition, Adjusted for ATP-III Criteria, c) New Definition, Adjusted for Strict Definition d) New Definition, Adjusted for HOMA-IR (NHANES-III n = 12 341; UK Biobank Participants Fasted <6h Excluded n = 41 431). **eFigure 2.** Adjusted HRs and 95% CIs for Risk of CVD Mortality in Subgroups Classified by BMI Categories and MH Defined by a) ATP-III Criteria; b) Strict Definition; c) HOMA-IR and Each Additionally Adjusted for Our New Exploratory Definition; (NHANES-III n = 12 341; UK Biobank Participants Fasted <6h Excluded n = 41 431; Definition With HOMA-IR Not Possible) **eFigure 3.** Adjusted HRs and 95% CIs for Risk of Total Mortality in Subgroups Classified by BMI Categories and MH Defined by a) Our New Exploratory Definition b) New Definition, Adjusted for ATP-III Criteria, c) New Definition, Adjusted for Strict Definition d) New Definition, Adjusted for HOMA-IR (NHANES-III n = 12 341; UK Biobank Participants Fasted <6h Excluded n = 41 431) **eFigure 4.** Adjusted HRs and 95% CIs for Risk of Total Mortality in Subgroups Classified by BMI Categories and MH Defined by a) ATP-III Criteria; b) Strict Definition; c) HOMA-IR and Each Additionally Adjusted for Our New Exploratory Definition; (NHANES-III n = 12 341; UK Biobank Participants Fasted <6h Excluded n = 41 431; Definition With HOMA-IR Not Possible) eReferences
