# Supplementary material for: An Empirically Derived Definition of Metabolically Healthy Obesity Based on Risk of Cardiovascular and Total Mortality
Source: JAMA Netw Open. 2021 May 7;4(5):e218505. doi: 10.1001/jamanetworkopen.2021.8505 (PMC8105750; doi:10.1001/jamanetworkopen.2021.8505)
Supplement: Supplement. — eTable 1. Baseline Assessment Within the Third National Health and Nutrition Examination Survey (NHANES-III) and the UK Biobank eTable 2. Summary of Missing Data for NHANES-III (n = 17 742) eTable 3. Cutoffs at Maximal Youden Index Among Obese Participants (NHANES-III n = 2511)* eTable 4. Baseline Characteristics of UK Biobank Participants in Groups of Metabolic Health and BMI (n = 374 079) eTable 5. Sensitivity Analysis of CVD Mortality eTable 6. Sensitivity Analysis of Total Mortality eTable 7. Stratified Analysis by Sex eTable 8. Stratified Analysis by CVD Type eTable 9. Adjusted HRs and 95% CIs of CVD and Total Mortality in Subgroups Classified by BMI Categories Extended by Obesity Class 1 to 3 and MH Defined by Our New Definition (UK Biobank n = 374 079) eTable 10. Adjusted HRs and 95% CIs of CVD and Total Mortality in Subgroups Classified by BMI Categories and MH Defined by a) Our New Definition, b) New Definition Without the Diabetes Criterion, c) New Definition Without the WHR Criterion, d) New Definition Without the Blood Pressure Criterion (UK Biobank n = 374 079) eFigure 1. Adjusted HRs and 95% CIs for Risk of CVD Mortality in Subgroups Classified by BMI Categories and MH Defined by a) Our New Exploratory Definition b) New Definition, Adjusted for ATP-III Criteria, c) New Definition, Adjusted for Strict Definition d) New Definition, Adjusted for HOMA-IR (NHANES-III n = 12 341; UK Biobank Participants Fasted <6h Excluded n = 41 431). eFigure 2. Adjusted HRs and 95% CIs for Risk of CVD Mortality in Subgroups Classified by BMI Categories and MH Defined by a) ATP-III Criteria; b) Strict Definition; c) HOMA-IR and Each Additionally Adjusted for Our New Exploratory Definition; (NHANES-III n = 12 341; UK Biobank Participants Fasted <6h Excluded n = 41 431; Definition With HOMA-IR Not Possible) eFigure 3. Adjusted HRs and 95% CIs for Risk of Total Mortality in Subgroups Classified by BMI Categories and MH Defined by a) Our New Exploratory Definition b) New Defini [file jamanetwopen-e218505-s001.pdf]

Supplementary Online Content

Zembic A, Eckel N, Stefan N, Baudry J, Schulze MB. An empirically derived definition of metabolically healthy obesity based on risk of cardiovascular and total mortality. *JAMA Netw Open*. 2021;4(5):e218505. doi:10.1001/jamanetworkopen.2021.8505

eTable 1. Baseline Assessment Within the Third National Health and Nutrition Examination Survey (NHANES-III) and the UK Biobank

eTable 2. Summary of Missing Data for NHANES-III (n=17,742)

eTable 3. Cutoffs at Maximal Youden Index Among Obese Participants (NHANES-III n=2,511)*

eTable 4. Baseline Characteristics of UK Biobank Participants in Groups of Metabolic Health and BMI (n= 374,079)

eTable 5. Sensitivity Analysis of CVD Mortality

eTable 6. Sensitivity Analysis of Total Mortality

eTable 7. Stratified Analysis by Sex

eTable 8. Stratified Analysis by CVD Type

eTable 9. Adjusted HRs and 95% CIs of CVD and Total Mortality in Subgroups Classified by BMI Categories Extended by Obesity Class 1 to 3 and MH Defined by Our New Definition (UK Biobank n=374,079)

eTable 10. Adjusted HRs and 95% CIs of CVD and Total Mortality in Subgroups Classified by BMI Categories and MH Defined by a) Our New Definition, b) New Definition Without the Diabetes Criterion, c) New Definition Without the WHR Criterion, d) New Definition Without the Blood Pressure Criterion (UK Biobank n=374,079)

eFigure 1. Adjusted HRs and 95% CIs for Risk of CVD Mortality in Subgroups Classified by BMI Categories and MH Defined by a) Our New Exploratory Definition b) New Definition, Adjusted for ATP-III Criteria, c) New Definition, Adjusted for Strict Definition d) New Definition, Adjusted for HOMA-IR (NHANES-III n=12 341; UK Biobank Participants Fasted <6h Excluded n=41,431).

eFigure 2. Adjusted HRs and 95% CIs for Risk of CVD Mortality in Subgroups Classified by BMI Categories and MH Defined by a) ATP-III Criteria; b) Strict Definition; c) HOMA-IR and Each Additionally Adjusted for Our New Exploratory Definition; (NHANES-III n=12,341; UK Biobank Participants Fasted <6h Excluded n=41,431; Definition With HOMA-IR Not Possible)

eFigure 3. Adjusted HRs and 95% CIs for Risk of Total Mortality in Subgroups Classified by BMI Categories and MH Defined by a) Our New Exploratory Definition b) New Definition, Adjusted for ATP-III Criteria, c) New Definition, Adjusted for Strict Definition d) New Definition, Adjusted for HOMA-IR (NHANES-III n=12,341; UK Biobank Participants Fasted <6h Excluded n=41,431)

eFigure 4. Adjusted HRs and 95% CIs for Risk of Total Mortality in Subgroups Classified by BMI Categories and MH Defined by a) ATP-III Criteria; b) Strict Definition; c) HOMA-IR and Each Additionally Adjusted for Our New Exploratory Definition; (NHANES-III n=12,341; UK Biobank Participants Fasted <6h Excluded n=41,431; Definition With HOMA-IR Not Possible)

eReferences

This supplementary material has been provided by the authors to give readers additional information about their work.

© 2021 Zembic A et al. *JAMA Network Open*

eTable 1: Baseline assessment within the third National Health and Nutrition Examination survey (NHANES-III) and the UK Biobank

	NHANES-III	UK Biobank
**Blood pressure**	Measured in sitting position;<br>up to six measurements were averaged (3 during examination, 3 during interview)	Measured in sitting position;<br>two automatic measurements, one minute apart. Additionally, two manual measurements if automatic was not possible
**Weight**	Measured to the nearest 0.1 kg with participants wearing underwear	Measured within 0.1 kg with participants wearing no shoes and heavy outer clothing; implausible values flagged immediately after input in assessment centre IT system
**Height**	Measured to the nearest 0.1 cm	Measured with participants wearing no shoes; implausible values flagged immediately after input in assessment centre IT system
**Waist circumference**	Measured at the midaxillary line of the body at minimal respiration to the nearest 0.1 cm	Measured at the level of the umbilicus
**Hip circumference**	Measured at the maximum extension of the buttock	Measured at hip level, using the same tape measure as for waist circumference
**Blood samples**	Taken via venepuncture	40-50 ml of blood taken via venepuncture
**Biomarkers available for the present analysis**	Triglycerides, total cholesterol, HDL-cholesterol, c-reactive protein, glucose, insulin, HbA1c, γ-glutamyltransferase, alanine-aminotransferase	Triglycerides, total cholesterol, HDL-cholesterol, glucose, HbA1c
**Fasting status**	Participants who had a morning examination were instructed to fast overnight and participants who had an afternoon or evening examination were instructed to fast 6 hours	Participants were not asked to fast before assessment; time since last meal was recorded as fasting time
**Prevalent diabetes**	Self-reported at the interview	Self-reported in questionnaire
**Current medication**	Participants were asked to bring the containers of current medication to the interview	Self-reported in questionnaire
**Lifestyle factors**	Self-reported at the interview	Self-reported in questionnaire and during interview
Ethnicity	Pre-defined answer options:<br>- Non-Hispanic white<br>- Non-Hispanic black<br>- Mexican-American<br>- Other	Pre-defined answer options:<br>- White<br>- Mixed<br>- Asian or Asian British<br>- Black or Black British<br>- Chinese<br>- Other ethnic group<br>- Do not know<br>- Prefer not to answer

NHANES-III: Baseline assessment included a computer assisted personal interview and physical examinations in a mobile examination centre or a home examination.[1–4]

UK Biobank: 22 assessment centres throughout the UK; participants had to fill out a self-administered questionnaire on a touch screen, then were asked further follow-up questions in a computer-assisted personal interview and had physical measurements taken.[5,6]

eTable 2: Summary of missing data for NHANES-III (n=17,742)*

Variable	Missing frequency<br>N (%)
**BMI**	63 (0.35)
**Sex**	0 (0)
**Age**	0 (0)
**Race**	0 (0)
**Years of school completed**	126 (0.71)
**Income**	308 (1.74)
**Marital status**	33 (0.18)
**Smoking status**	1 (0.01)
**Alcohol consumption**	44 (0.25)
**Physical activity**	45 (0.25)
**Waist circumference**	1,329 (7.49)
**Waist-to-hip ratio**	1,354 (7.63)
**Systolic blood pressure**	493 (2.78)
**Diastolic blood pressure**	502 (2.83)
**Triglycerides**	1,057 (5.95)
**Total cholesterol**	1,020 (5.75)
**HDL cholesterol**	1,137 (6.40)
**Fasting glucose**	1,875 (10.56)
**HbA1c**	840 (4.73)
**C-reactive protein**	1,155 (6.51)
**Alanine-aminotransferase**	1,256 (7.08)
**γ-glutamyltransferase**	4,952 (27.90)
**Lipid-lowering medication**	15 (0.08)
**Blood pressure-lowering medication**	15 (0.08)
**Self-reported diabetes**	24 (0.14)

*Exclusion of participants who did not complete examination and not eligible for follow-up (younger than 16 years).

© 2021 Zembic A et al. *JAMA Network Open*

eTable 3: Cutoffs at maximal Youden Index among obese participants (NHANES-III n=2,511)*

	CVD mortality		Total mortality	
	**Youden Index**	**Cut-off**	**Youden Index**	**Cut-off**
**Systolic blood pressure**	0.460	125	0.320	124
**Waist-to-hip ratio**				
Women	0.338	0.95	0.208	0.92
Men	0.291	1.03	0.223	1.03

*Exclusion of participants with blood pressure-lowering, glucose-lowering or lipid-lowering medication.

© 2021 Zembic A et al. *JAMA Network Open*

eTable 4: Baseline characteristics of UK Biobank participants in groups of metabolic health and BMI (n= 374,079)

	MHNW	MUHNW	MHOW	MUHOW	MHO	MUHO
n (%)	60,516 (16.2)	65,082 (17.4)	49,227 (13.2)	110,897 (29.7)	17,076 (4.6)	71,281 (19.1)
CVD mortality n (%)	109 (0.18)	411 (0.63)	114 (0.23)	796 (0.72)	39 (0.23)	693 (0.97)
Total mortality n (%)	1,273 (2.10)	2,533 (3.89)	1,038 (2.11)	4,415 (3.98)	387 (2.27)	3,304 (4.64)
Women [%]	72.4	59.2	58.5	42.8	68.4	50.2
Mean age (Standard Deviation)	52.7 (7.91)	58.1 (7.58)	53.2 (7.94)	58.2 (7.60)	52.3 (7.73)	57.4 (7.61)
Mean BMI (Standard Deviation)	22.6 (1.58)	23.1 (1.45)	27.1 (1.38)	27.4 (1.40)	33.3 (3.41)	34.0 (3.94)
**Ethnicity [%]**						
White	95.1	95.7	94.6	95.5	93.3	95.0
Mixed	0.80	0.45	0.70	0.43	0.84	0.53
Asian or Asian British	1.72	1.82	2.06	1.86	1.80	1.50
Black or Black British	0.77	0.75	1.34	1.25	2.66	2.09
Chinese	0.69	0.51	0.28	0.20	0.07	0.07
Other	0.95	0.61	0.99	0.76	1.30	0.85
**Education [%]**						
University/college	45.0	35.4	36.5	30.0	30.2	24.1
A levels	12.8	11.7	12.5	10.5	12.1	10.3
O levels/GCSE	20.2	21.0	22.1	21.4	23.7	22.2
CSE	5.23	4.39	6.52	4.99	8.23	6.22
NVQ	4.10	5.53	5.81	7.54	6.69	8.05
Other professional	4.07	5.33	4.77	5.45	4.93	5.75
**Income [%]**						
< 18,000 GBP	13.5	18.9	14.6	19.3	18.7	23.3
**Marital status [%]**						
Married and living together	71.6	73.7	73.8	76.3	68.6	70.9
Living alone	17.6	19.4	16.4	17.2	17.9	20.3
Living with other family member	10.8	6.97	9.80	6.52	13.5	8.76
**Smoking [%]**						
Never	60.5	58.7	56.5	53.4	55.0	51.8
Former	28.1	30.6	32.1	37.1	33.2	39.1
Current	11.4	10.8	11.4	9.51	11.9	9.05
**Alcohol [%]**						
Never	7.11	6.81	7.14	6.72	9.62	9.46
Special occasions only	10.0	9.49	10.9	9.53	16.4	15.2
1–3 times per month	11.7	9.07	12.4	9.71	16.6	12.9
1–2 times per week	26.7	24.1	28.5	25.4	28.4	26.1
3–4 times per week	25.0	24.8	23.9	25.2	17.9	19.8
Daily or almost daily	19.5	25.7	17.2	23.5	11.2	16.6
**Physical activity [%]**						
Never	33.5	36.3	37.2	39.5	47.6	50.1
≤20 minutes of vigorous activity per week	12.7	12.3	12.3	12.2	11.7	11.5
>20 minutes of vigorous activity per week	53.9	51.3	50.5	48.4	40.8	38.4

MHNW: metabolically healthy normal-weight, MUHNW: metabolically unhealthy normal-weight, MHOW: metabolically healthy overweight, MUHOW: metabolically unhealthy overweight, MHO: metabolically healthy obese, MUHO: metabolically unhealthy obese.

© 2021 Zembic A et al. *JAMA Network Open*

eTable 5: Sensitivity analysis of CVD Mortality: Adjusted hazard ratios (HRs) and 95% confidence intervals (CI) of cardiovascular disease (CVD) mortality in subgroups classified by BMI categories and metabolic health (MH) defined by our new definition a) among never smokers; b) excluding deaths within first two years of follow-up; c) excluding participants >60 years of age; d) changing the reference category from normal weight to BMI 20.0-22.49 kg/m²

	n	HR	95% CI	n	HR	95% CI	n	HR	95% CI	n	HR	95% CI
**NHANES-III**	**a) Never smokers (n = 6,253)**			**b) Excluding deaths first 2 years follow-up (n = 12,205)**			**c) Excluding participants >60 (n = 9,928)**			**d) Reference category BMI 20.0-22.49 kg/m² (n = 11,427)**		
**MHNW**	2,042	1 (Ref)		3,755	1 (Ref)		3,544	1 (Ref)		1,440	1 (Ref)	
**MUHNW**	417	2.69	0.95 - 7.56	1,073	3.59	2.06 - 6.25	527	6.02	2.51 - 14.4	376	4.08	2.15 - 7.72
**MHOW**	1,242	0.55	0.16 - 1.96	2,339	1.11	0.60 - 2.06	2,201	1.51	0.53 - 4.32	4,149	0.92	0.53 - 1.61
**MUHOW**	813	2.40	0.92 - 6.26	1,830	2.41	1.38 - 4.22	1,045	3.99	1.64 - 9.74	2,526	2.21	1.16 - 4.22
**MHO**	720	0.41	0.11 - 1.51	1,229	0.72	0.31 - 1.65	1,178	1.15	0.50 - 2.63	1,235	0.59	0.24 - 1.45
**MUHO**	1,019	2.68	1.07 - 6.69	1,979	3.67	2.00 - 6.73	1,388	6.96	2.78 - 17.5	2,001	3.17	1.61 - 6.24
**UK Biobank**	**a) Never smokers (n = 208,139)**			**b) Excluding deaths first 2 years follow-up (n = 372,578)**			**c) Excluding participants >60 (n = 236,597)**			**d) Reference category BMI 20.0-22.49 kg/m² (n = 367,101)**		
**MHNW**	36,610	1 (Ref)		60,338	1 (Ref)		48,197	1 (Ref)		21,263	1 (Ref)	
**MUHNW**	38,171	2.18	1.54 - 3.07	64,768	2.10	1.66 - 2.65	35,295	2.46	1.76 - 3.44	17,866	2.19	1.48 - 3.23
**MHOW**	27,809	1.30	0.86 - 1.98	49,114	1.09	0.81 - 1.45	38,164	1.00	0.67 - 1.51	84,184	1.15	0.79 - 1.67
**MUHOW**	59,219	2.24	1.61 - 3.12	110,412	2.03	1.62 - 2.54	59,067	2.34	1.70 - 3.21	155,431	2.15	1.51 - 3.04
**MHO**	9,386	1.31	0.71 - 2.40	17,025	1.29	0.88 - 1.91	13,909	1.4	0.84 - 2.32	17,076	1.28	0.81 - 2.04
**MUHO**	36,944	3.29	2.36 - 4.59	70,921	2.90	2.31 - 3.64	41,965	3.65	2.67 - 5.01	71,281	3.02	2.12 - 4.29

MHNW: metabolically healthy normal-weight, MUHNW: metabolically unhealthy normal-weight, MHOW: metabolically healthy overweight, MUHOW: metabolically unhealthy overweight, MHO: metabolically healthy obese, MUHO: metabolically unhealthy obese. HRs and 95% CIs are weighted to the US population in NHANES-III. HRs adjusted for age, sex, ethnicity, education, income, marital status, smoking status, alcohol consumption, physical activity and assessment centre (assessment centre UK Biobank only).

eTable 6: Sensitivity analysis of Total Mortality: Adjusted HRs and 95% CIs of Total mortality in subgroups classified by BMI categories and MH defined by our new definition a) among never smokers; b) excluding deaths within first two years of follow-up; c) excluding participants >60 years of age; d) changing the reference category from normal weight to BMI 20.0-22.49 kg/m²

	n	HR	95% CI	n	HR	95% CI	n	HR	95% CI	n	HR	95% CI
**NHANES-III**	**a) Never smokers (n = 6,253)**			**b) Excluding deaths first 2 years follow-up (n = 12,205)**			**c) Excluding participants >60 (n = 9,928)**			**d) Reference category BMI 20.0-22.49 kg/m² (n = 11,427)**		
**MHNW**	2,042	1 (Ref)		3,755	1 (Ref)		3,544	1 (Ref)		1,440	1 (Ref)	
**MUHNW**	417	1.53	0.92 - 2.52	1,073	2	1.44 - 2.49	527	1.9	1.18 - 3.08	376	1.74	1.27 - 2.37
**MHOW**	1,242	1.14	0.60 - 2.17	2,339	1.12	0.80 - 1.57	2,201	1.05	0.68 - 1.60	4,149	0.82	0.62 - 1.08
**MUHOW**	813	1.42	0.86 - 2.37	1,830	1.5	1.14 - 1.98	1,045	1.74	1.19 - 2.53	2,526	1.21	0.90 - 1.60
**MHO**	720	1.16	0.51 - 2.65	1,229	1.08	0.73 - 1.60	1,178	1.06	0.61 - 1.83	1,235	0.84	0.55 - 1.29
**MUHO**	1,019	1.79	1.07 - 2.99	1,979	1.94	1.45 - 2.59	1,388	2.36	1.45 - 2.59	2,001	1.5	1.09 - 2.06
**UK Biobank**	**a) Never smokers (n = 208,139)**			**b) Excluding deaths first 2 years follow-up (n = 372,578)**			**c) Excluding participants >60 (n = 236,597)**			**d) Reference category BMI 20.0-22.49 kg/m² (n = 367,101)**		
**MHNW**	36,610	1 (Ref)		60,338	1 (Ref)		48,197	1 (Ref)		21,263	1 (Ref)	
**MUHNW**	38,171	1.10	0.99 - 1.23	64,768	1.14	1.06 - 1.23	35,295	1.21	1.10 - 1.34	17,866	1.19	1.06 - 1.34
**MHOW**	27,809	0.96	0.85 - 1.09	49,114	0.92	0.85 - 1.01	38,164	0.87	0.78 - 0.97	84,184	0.87	0.78 - 0.97
**MUHOW**	59,219	1.14	1.03 - 1.26	110,412	1.08	1.01 - 1.16	59,067	1.03	0.93 - 1.13	155,431	1.01	0.92 - 1.12
**MHO**	9,386	1.12	0.94 - 1.34	17,025	0.99	0.88 - 1.13	13,909	0.98	0.84 - 1.14	17,076	0.94	0.82 - 1.08
**MUHO**	36,944	1.43	1.29 - 1.58	70,921	1.28	1.20 - 1.38	41,965	1.36	1.23 - 1.49	71,281	1.19	1.08 - 1.32

MHNW: metabolically healthy normal-weight, MUHNW: metabolically unhealthy normal-weight, MHOW: metabolically healthy overweight, MUHOW: metabolically unhealthy overweight, MHO: metabolically healthy obese, MUHO: metabolically unhealthy obese. HRs and 95% CIs are weighted to the US population in NHANES-III. HRs adjusted for age, sex, ethnicity, education, income, marital status, smoking status, alcohol consumption, physical activity and assessment centre (assessment centre UK Biobank only).

© 2021 Zembic A et al. *JAMA Network Open*

eTable 7: Stratified analysis by sex

	n	HR	95% CI	n	HR	95% CI		HR	95% CI	HR	95% CI
**NHANES-III**	**CVD mortality women (n = 6,527)**			**CVD mortality men (n = 5,816)**				**Total mortality women (n = 6,527)**		**Total mortality men (n = 5,816)**	
**MHNW**	2,048	1 (Ref)		1,733	1 (Ref)			1 (Ref)		1 (Ref)	
**MUHNW**	524	2.49	0.99 - 6.26	583	5.37	2.58 - 11.2		2.33	1.55 - 3.50	1.54	1.06 - 2.25
**MHOW**	1,082	0.81	0.30 - 2.19	1,263	1.55	0.53 - 4.56		1.61	1.05 - 2.47	0.84	0.51 - 1.38
**MUHOW**	855	2.07	0.89 - 4.78	1,018	3.37	1.70 - 6.69		2.08	1.46 - 2.98	1.24	0.89 - 1.72
**MHO**	840	0.50	0.14 - 1.77	396	1.07	0.32 - 3.56		1.16	0.67 - 2.01	1.02	0.57 - 1.82
**MUHO**	1,178	3.29	1.25 - 8.64	823	4.72	2.11 - 10.5		2.55	1.72 - 3.76	1.53	0.97 - 2.41
**UK Biobank**	**CVD mortality women (n = 205,972)**			**CVD mortality men (n = 168,107)**				**Total mortality women (n = 205 972)**		**Total mortality men (n = 168 107)**	
**MHNW**	43,813	1 (Ref)		16,703	1 (Ref)			1 (Ref)		1 (Ref)	
**MUHNW**	38,521	2.32	1.66 - 3.23	26,561	1.72	1.30 - 2.28		1.13	1.03 - 1.24	1.14	1.03 - 1.26
**MHOW**	28,807	1.11	0.73 - 1.71	20,420	1.06	0.76 - 1.49		0.94	0.84 - 1.05	0.85	0.75 - 0.96
**MUHOW**	47,404	2.31	1.67 - 3.20	63,493	1.78	1.36 - 2.32		1.13	1.03 - 1.24	1.01	0.92 - 1.12
**MHO**	11,678	0.97	0.54 - 1.78	5,398	1.33	0.83 - 2.12		1.01	0.87 - 1.17	1.00	0.81 - 1.16
**MUHO**	35,749	2.63	1.89 - 3.65	35,532	2.67	2.04 - 3.50		1.27	1.16 - 1.40	1.23	1.11 - 1.35

MHNW: metabolically healthy normal-weight, MUHNW: metabolically unhealthy normal-weight, MHOW: metabolically healthy overweight, MUHOW: metabolically unhealthy overweight, MHO: metabolically healthy obese, MUHO: metabolically unhealthy obese. HRs and 95% CIs are weighted to the US population in NHANES-III. HRs adjusted for age, sex, ethnicity, education, income, marital status, smoking status, alcohol consumption, physical activity and assessment centre (assessment centre UK Biobank only).

© 2021 Zembic A et al. *JAMA Network Open*

eTable 8: Stratified analysis by CVD type: Adjusted HRs and 95% CIs of mortality from coronary heart disease (CHD) and stroke in subgroups classified by BMI categories and MH defined by our new definition

	n	HR	95% CI		HR	95% CI
**NHANES-III**		**CHD (n = 12,341)**			**Stroke (n = 12,341)**	
**MHNW**	3,781	1 (Ref)			1 (Ref)	
**MUHNW**	1,107	3.97	1.71 - 9.24		1.25	0.45 - 3.94
**MHOW**	2,344	1.28	0.62 - 2.63		0.52	0.13 - 2.08
**MUHOW**	1,873	2.46	1.24 - 4.88		1.19	0.37 - 3.87
**MHO**	1,235	0.43	0.11 - 1.75		0.23	0.55 - 0.93
**MUHO**	2,001	4.30	1.93 - 9.57		1.46	0.46 - 4.65
**UK Biobank**		**CHD (n = 374,079)**			**Stroke (n = 374,079)**	
**MHNW**	60,516	1 (Ref)			1 (Ref)	
**MUHNW**	65,082	2.51	1.83 - 3.46		1.90	1.27 - 2.83
**MHOW**	49,227	1.34	0.91 - 1.97		0.75	0.43 - 1.30
**MUHOW**	110,897	3.15	2.33 - 4.27		1.76	1.20 - 2.58
**MHO**	17,076	1.22	0.70 - 2.12		0.59	0.25 - 1.42
**MUHO**	71,281	4.39	3.23 - 5.86		1.82	1.22 - 2.70

MHNW: metabolically healthy normal-weight, MUHNW: metabolically unhealthy normal-weight, MHOW: metabolically healthy overweight, MUHOW: metabolically unhealthy overweight, MHO: metabolically healthy obese, MUHO: metabolically unhealthy obese. HRs and 95% CIs are weighted to the US population in NHANES-III. HRs adjusted for age, sex, ethnicity, education, income, marital status, smoking status, alcohol consumption, physical activity and assessment centre (assessment centre UK Biobank only).

© 2021 Zembic A et al. *JAMA Network Open*

eTable 9: Adjusted HRs and 95% CIs of CVD- and Total mortality in subgroups classified by BMI categories extended by obesity class 1 to 3 and MH defined by our new definition (UK Biobank n=374,079)

	CVD mortality				Total mortality		
	n	n(deaths)	HR	95% CI	n(deaths)	HR	95% CI
**MHNW**	60,516	109	1	-	1,273	1	-
**MUHNW**	65,082	411	1.94	1.57 - 2.41	2,533	1.12	1.05 - 1.20
**MHOW**	49,227	114	1.10	0.84 - 1.43	1,038	0.89	0.82 - 0.96
**MUHOW**	110,897	796	1.94	1.58 - 2.39	4,415	1.04	0.98 - 1.11
**MHO class 1**	13,300	27	1.00	0.65 - 1.52	294	0.93	0.82 - 1.06
**MUHO class 1**	50,537	440	2.39	1.93 - 2.97	2,199	1.14	1.06 - 1.22
**MHO class 2**	2,899	4	0.83	0.31 - 2.26	68	1.11	0.87 - 1.42
**MUHO class 2**	14,853	172	3.45	2.70 - 4.41	738	1.37	1.25 - 1.50
**MHO class 3**	877	8	6.34	3.08 - 13.02	25	1.45	0.98 - 2.16
**MUHO class 3**	5,891	81	4.60	3.43 - 6.16	367	1.80	1.60 - 2.03

MHNW: metabolically healthy normal-weight, MUHNW: metabolically unhealthy normal-weight, MHOW: metabolically healthy overweight, MUHOW: metabolically unhealthy overweight, MHO: metabolically healthy obese, MUHO: metabolically unhealthy obese. Obesity class 1: BMI 30.0–34.9kg/m², obesity class 2: BMI 35.0–39.9kg/m², obesity class 3: BMI ≥40kg/m². HRs adjusted for age, sex, ethnicity, education, income, marital status, smoking status, alcohol consumption, physical activity and assessment centre.

© 2021 Zembic A et al. *JAMA Network Open*

eTable 10: Adjusted HRs and 95% CIs of CVD- and Total mortality in subgroups classified by BMI categories and MH defined by a) our new definition, b) new definition without the diabetes criterion, c) new definition without the WHR criterion, d) new definition without the blood pressure criterion (UK Biobank n=374,079)

	n(deaths)	HR	95% CI	n(deaths)	HR	95% CI	n(deaths)	HR	95% CI	n(deaths)	HR	95% CI
**CVD Mortality**	**a) Main analysis**			**b) Without diabetes criterion**			**c) Without WHR criterion**			**d) Without BP criterion**		
**MHNW**	109	1 (Ref)		110	1 (Ref)		110	1 (Ref)		479	1 (Ref)	
**MUHNW**	411	1.96	1.58 - 2.43	410	2.00	1.61 - 2.47	410	1.96	1.58 - 2.42	41	1.97	1.42 - 2.74
**MHOW**	114	1.10	0.85 - 1.43	117	1.11	0.85 - 1.44	120	1.12	0.86 - 1.45	767	1.06	0.94 - 1.19
**MUHOW**	796	1.97	1.60 - 2.41	793	1.99	1.62 - 2.44	790	1.96	1.60 - 2.41	143	1.80	1.48 - 2.17
**MHO**	39	1.17	0.81 - 1.69	46	1.31	0.92 - 1.84	50	1.28	0.92 - 1.80	2,416	1.32	1.15 - 1.51
**MUHO**	693	2.76	2.25 - 3.40	3,276	2.79	2.27 - 3.43	682	2.77	2.25 - 3.40	1,275	2.44	2.10 - 2.83
**Total mortality**	**a) Main analysis**			**b) Without diabetes criterion**			**c) Without WHR criterion**			**d) Without BP criterion**		
**MHNW**	1,273	1 (Ref)		1,306	1 (Ref)		1,290	1 (Ref)		3,580	1 (Ref)	
**MUHNW**	2,533	1.13	1.05 - 1.21	2,500	1.11	1.04 - 1.19	2,516	1.11	1.04 - 1.19	226	1.77	1.55 - 2.03
**MHOW**	1,083	0.89	0.82 - 0.96	1,074	0.89	0.82 - 0.96	1,082	0.89	0.82 - 0.96	4,743	0.92	0.88 - 0.97
**MUHOW**	4,415	1.05	0.98 - 1.12	4,379	1.04	0.97 - 1.10	4,371	1.04	0.97 - 1.11	710	1.36	1.26 - 1.48
**MHO**	387	0.98	0.87 - 1.10	415	0.98	0.88 - 1.10	456	1.00	0.90 - 1.12	415	1.03	0.98 - 1.09
**MUHO**	3,304	1.24	1.16 - 1.32	686	1.23	1.15 - 1.31	3,235	1.23	1.15 - 1.31	317	1.48	1.38 - 1.58

MHNW: metabolically healthy normal-weight, MUHNW: metabolically unhealthy normal-weight, MHOW: metabolically healthy overweight, MUHOW: metabolically unhealthy overweight, MHO: metabolically healthy obese, MUHO: metabolically unhealthy obese. HRs adjusted for age, sex, ethnicity, education, income, marital status, smoking status, alcohol consumption, physical activity and assessment centre.

© 2021 Zembic A et al. *JAMA Network Open*

eFigure 1: Adjusted HRs and 95% CIs for risk of CVD mortality in subgroups classified by BMI categories and MH defined by a) our new exploratory definition b) new definition, adjusted for ATP-III criteria, c) new definition, adjusted for strict definition d) new definition, adjusted for HOMA-IR (NHANES-III n=12 341; UK Biobank participants fasted <6h excluded n=41,431).

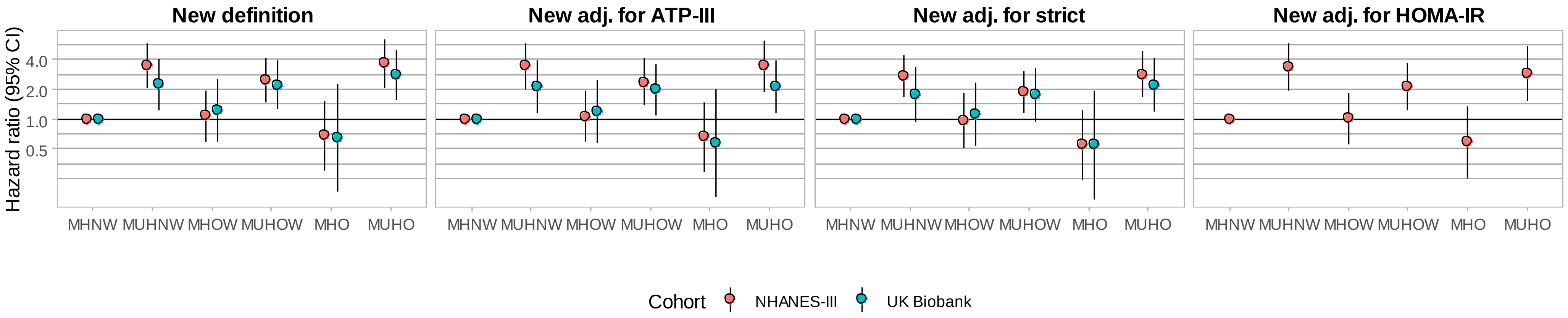

MHNW: metabolically healthy normal-weight, MUHNW: metabolically unhealthy normal-weight, MHOW: metabolically healthy overweight, MUHOW: metabolically unhealthy overweight, MHO: metabolically healthy obese, MUHO: metabolically unhealthy obese. HRs adjusted for age, sex, ethnicity, education, income, marital status, smoking status, alcohol consumption, physical activity and assessment centre (assessment centre UK Biobank only).

© 2021 Zembic A et al. *JAMA Network Open*

eFigure 2: Adjusted HRs and 95% CIs for risk of CVD mortality in subgroups classified by BMI categories and MH defined by a) ATP-III criteria; b) Strict definition; c) HOMA-IR and each additionally adjusted for our new exploratory definition; (NHANES-III n=12,341; UK Biobank participants fasted <6h excluded n=41,431; definition with HOMA-IR not possible)

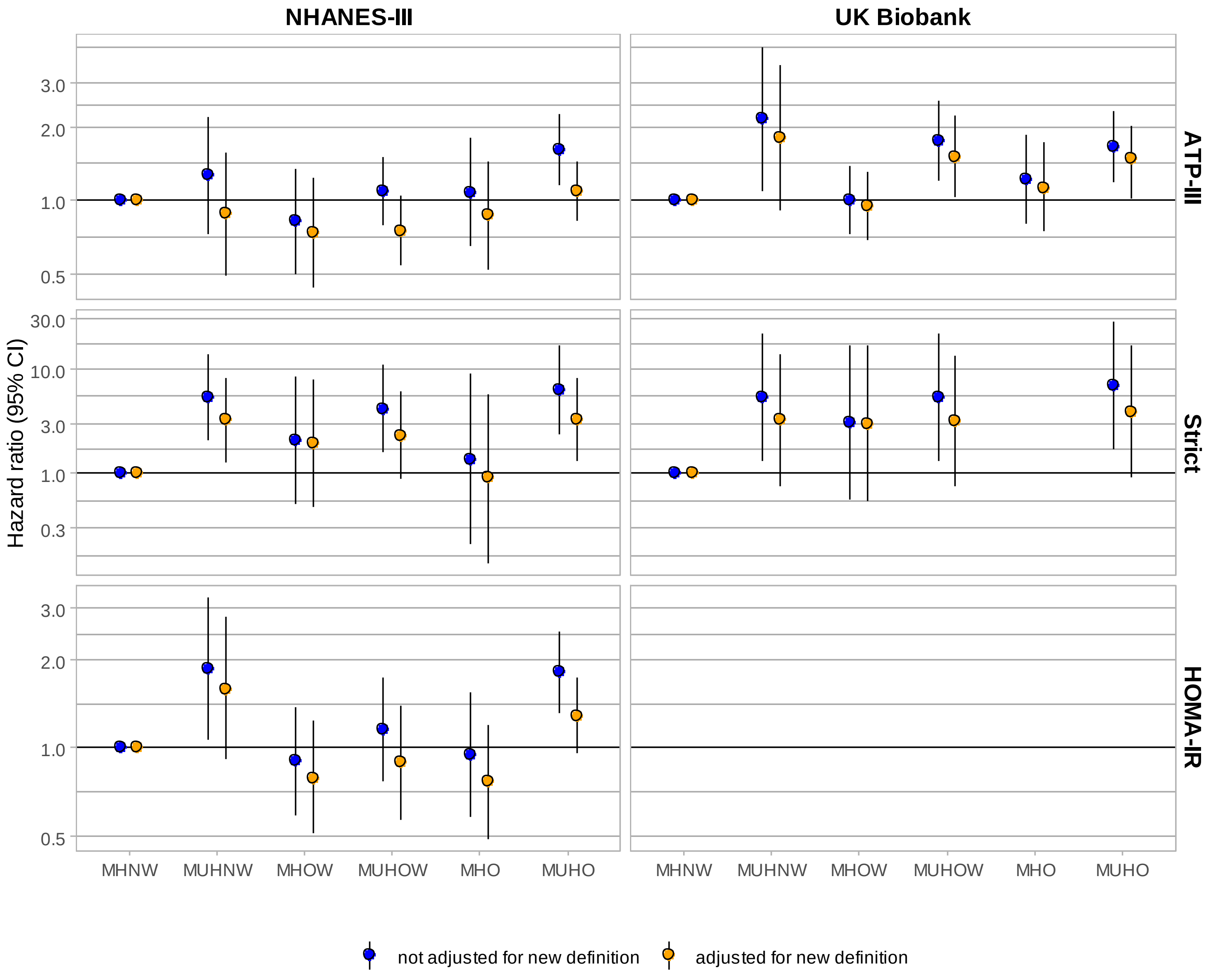

MHNW: metabolically healthy normal-weight, MUHNW: metabolically unhealthy normal-weight, MHOW: metabolically healthy overweight, MUHOW: metabolically unhealthy overweight, MHO: metabolically healthy obese, MUHO: metabolically unhealthy obese. No cases in the MHO group strict definition UK Biobank. HRs adjusted for age, sex, ethnicity, education, income, marital status, smoking status, alcohol consumption, physical activity and assessment centre (assessment centre UK Biobank only).

© 2021 Zembic A et al. *JAMA Network Open*

eFigure 3: Adjusted HRs and 95% CIs for risk of Total mortality in subgroups classified by BMI categories and MH defined by a) our new exploratory definition b) new definition, adjusted for ATP-III criteria, c) new definition, adjusted for strict definition d) new definition, adjusted for HOMA-IR (NHANES-III n=12,341; UK Biobank participants fasted <6h excluded n=41,431).

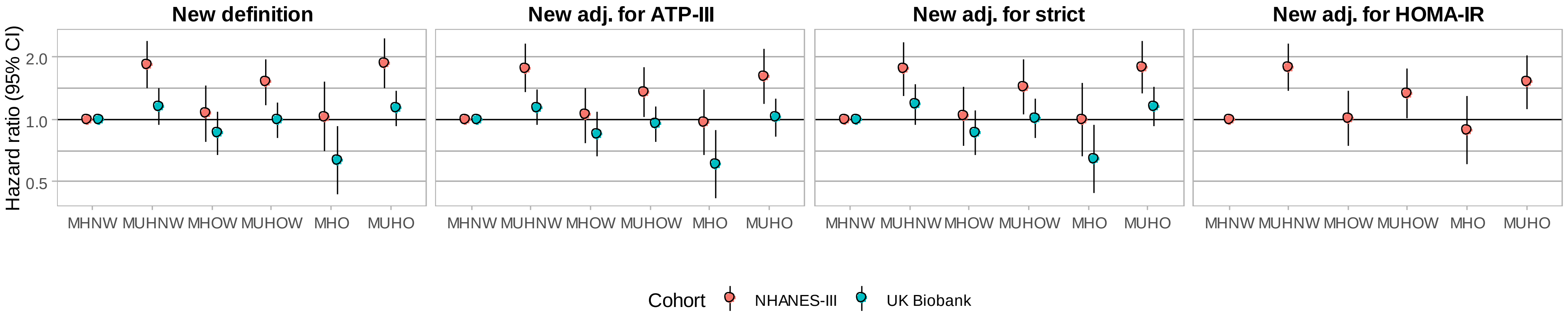

MHNW: metabolically healthy normal-weight, MUHNW: metabolically unhealthy normal-weight, MHOW: metabolically healthy overweight, MUHOW: metabolically unhealthy overweight, MHO: metabolically healthy obese, MUHO: metabolically unhealthy obese. HRs adjusted for age, sex, ethnicity, education, income, marital status, smoking status, alcohol consumption, physical activity and assessment centre (assessment centre UK Biobank only).

© 2021 Zembic A et al. *JAMA Network Open*

eFigure 4: Adjusted HRs and 95% CIs for risk of Total mortality in subgroups classified by BMI categories and MH defined by a) ATP-III criteria; b) Strict definition; c) HOMA-IR and each additionally adjusted for our new exploratory definition; (NHANES-III n=12,341; UK Biobank participants fasted <6h excluded n=41,431; definition with HOMA-IR not possible)

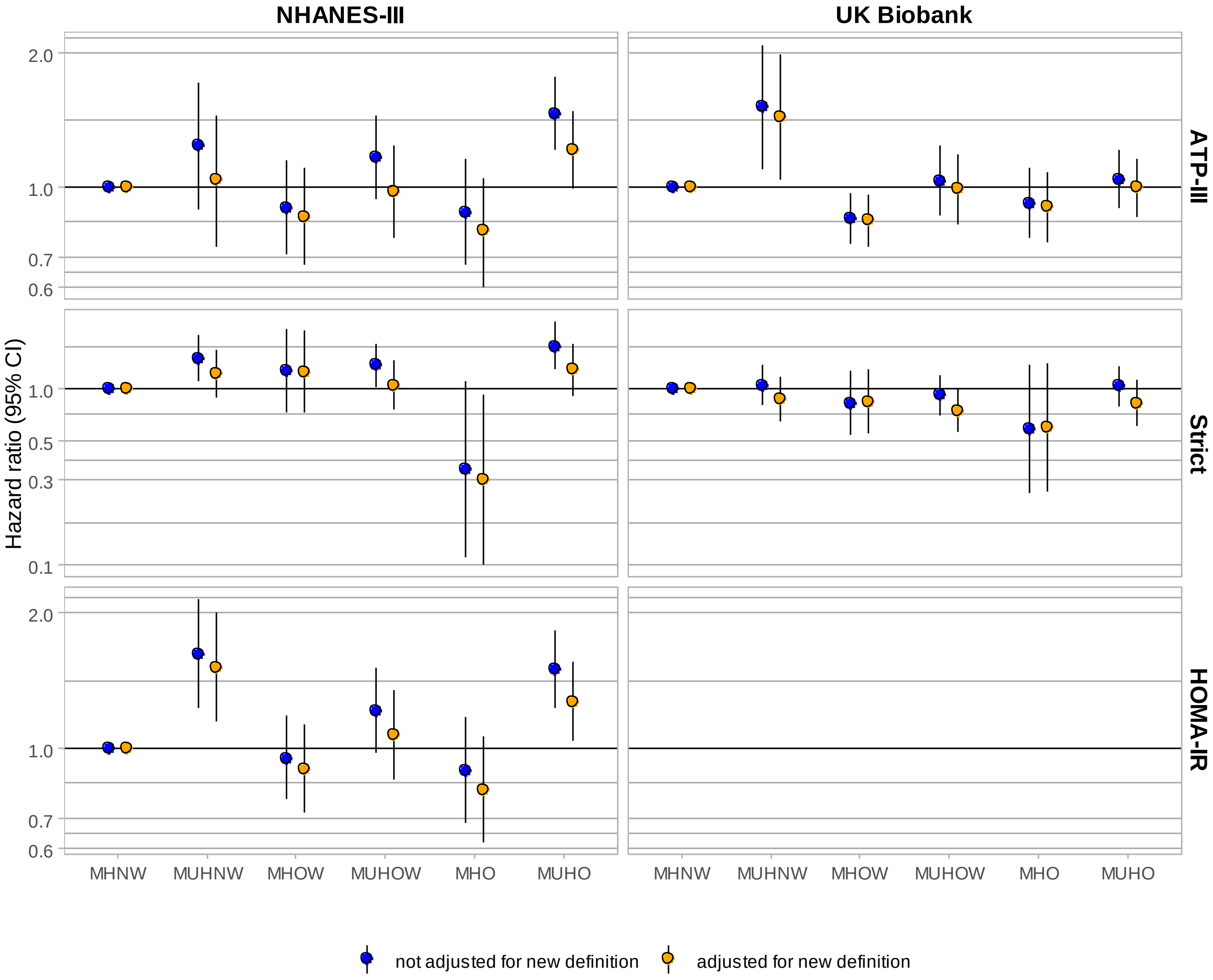

MHNW: metabolically healthy normal-weight, MUHNW: metabolically unhealthy normal-weight, MHOW: metabolically healthy overweight, MUHOW: metabolically unhealthy overweight, MHO: metabolically healthy obese, MUHO: metabolically unhealthy obese. HRs adjusted for age, sex, ethnicity, education, income, marital status, smoking status, alcohol consumption, physical activity and assessment centre (assessment centre UK Biobank only).

© 2021 Zembic A et al. *JAMA Network Open*

eReferences

1 National Center for Health Statistics. Plan and operation of the Third National Health and Nutrition Examination Survey, 1988-94. Series 1: programs and collection procedures. 1994 http://www.ncbi.nlm.nih.gov/pubmed/7975354.

2 National Center for Health Statistics. National Health and Nutrition Examination Survey NHANES III: Body Measurements (Anthropometry). 1988 https://wwwn.cdc.gov/nchs/data/nhanes3/manuals/anthro.pdf.

3 Gunter EW, Lewis BG, Koncikowski SM. Laboratory procedures used for the Third National Health and Nutrition Examination Survey (NHANES-III), 1988–1994. 1996 https://www.cdc.gov/nchs/data/nhanes/nhanes3/cdrom/NCHS/MANUALS/LABMAN.PDF.

4 National Center for Health Statistics. NHANES 2005-2006: Prescription Medications Data Documentation, Codebook, and Frequencies. 2009 https://wwwn.cdc.gov/Nchs/Nhanes/2005-2006/RXQ_RX_D.htm.

5 UK Biobank. Protocol for a large-scale prospective epidemiological resource. 2007 https://www.ukbiobank.ac.uk/wp-content/uploads/2011/11/UK-Biobank-Protocol.pdf.

6 UK Biobank. Blood pressure measurement. 2011 https://biobank.ctsu.ox.ac.uk/crystal/crystal/docs/Bloodpressure.pdf.

© 2021 Zembic A et al. *JAMA Network Open*
